# 

**Published:** 1875-02

**Authors:** 


### Contents of February Number.

### ORIGINAL DEPARTMENT.

ART. I.—Trichina Spiralis and Trichiniasis. By Geo. E. Blackham, M. D. .... 241

ART. II.—Abstract of the Proceedings of the Buffalo Medical Association. Reported by Joseph Fowler, M. D., Secretary, pro tem.... 252

ART. III.—Report of Surgical Cases at the Buffalo General Hospital, with Clinical Remarks. By Prof. J. F. Miner, M. D. Reported by B. Bartow, M. D., and W. W. Miner, M. D., .... 258

### MISCELLANEOUS.

Medical Society of the State of New York. Sixty-Ninth Annual Meeting, .... 262

### EDITORIAL DEPARTMENT.

Twenty-Ninth Annual Commencement of the Buffalo Medical College.. 277

Items, .... 278

Books Reviewed:

Ziemssen's Cyclopædia of the Practice of Medicine, .... 279

Books and Pamphlets Received .... 280

THE BUFFALO

